# Down-Regulation of CXXC5 De-Represses MYCL1 to Promote Hepatic Stellate Cell Activation

**DOI:** 10.3389/fcell.2021.680344

**Published:** 2021-09-21

**Authors:** Xiaoyan Wu, Wenhui Dong, Ming Kong, Haozhen Ren, Jinglin Wang, Longcheng Shang, Zhengyi Zhu, Wei Zhu, Xiaolei Shi

**Affiliations:** ^1^Department of Hepatobiliary Surgery, Affiliated Nanjing Drum Tower Hospital of Nanjing University Medical School, Nanjing, China; ^2^Hepatobiliary Institute of Nanjing University, Nanjing, China; ^3^Institute of Biomedical Research, Liaocheng University, Liaocheng, China; ^4^Key Laboratory of Targeted Intervention of Cardiovascular Disease, Collaborative Innovation Center for Cardiovascular Translational Medicine, and Center for Experimental Medicine, Department of Pathophysiology, Nanjing Medical University, Nanjing, China; ^5^Department of Anesthesiology, The Affiliated Drum Tower Hospital of Nanjing University Medical School, Nanjing, China

**Keywords:** transcriptional regulation, epigenetics, transcription repressor, liver fibrosis, hepatic stellate cells, DNA methylation

## Abstract

Liver fibrosis is mediated by myofibroblasts, a specialized cell type involved in wound healing and extracellular matrix production. Hepatic stellate cells (HSC) are the major source of myofibroblasts in the fibrotic livers. In the present study we investigated the involvement of CXXC-type zinc-finger protein 5 (CXXC5) in HSC activation and the underlying mechanism. Down-regulation of CXXC5 was observed in activated HSCs compared to quiescent HSCs both *in vivo* and *in vitro*. In accordance, over-expression of CXXC5 suppressed HSC activation. RNA-seq analysis revealed that CXXC5 influenced multiple signaling pathways to regulate HSC activation. The proto-oncogene MYCL1 was identified as a novel target for CXXC5. CXXC5 bound to the proximal MYCL1 promoter to repress MYCL1 transcription in quiescent HSCs. Loss of CXXC5 expression during HSC activation led to the removal of CpG methylation and acquisition of acetylated histone H3K9/H3K27 on the MYCL1 promoter resulting in MYCL1 trans-activation. Finally, MYCL1 knockdown attenuated HSC activation whereas MYCL1 over-expression partially relieved the blockade of HSC activation by CXXC5. In conclusion, our data unveil a novel transcriptional mechanism contributing to HSC activation and liver fibrosis.

## Introduction

Liver fibrosis is a key intermediate step in such irreversible liver diseases as hepatocellular carcinoma and cirrhosis (Luedde and Schwabe, 2011). Liver fibrosis is mediated primarily by myofibroblasts, a morphologically and functionally distinctive cell type specialized in the synthesis of extracellular matrix (ECM) proteins (Lee Y.A. et al., 2015). Myofibrobalsts are typically undetectable under physiological conditions but undergo massive expansion during liver fibrogenesis through a combination of trans-differentiation and proliferation (Tsuchida and Friedman, 2017). The origins of myofibroblasts in the fibrotic livers were once a subject matter of great controversy and an array of cells, including hepatic parenchymal cells, sinusoidal endothelial cells, portal fibroblasts, and oval cells, were considered as potential predecessors to mature myofibroblasts (Kisseleva, 2017). Recently, Mederacke et al. (2013) have tracked the fate of emerging myofibroblasts in liver fibrosis using the lineage-tracing technique and discovered that this unique population of cells are invariably derived from hepatic stellate cells tucked between the sinusoidal endothelium and the parenchyma. Absent of liver injury, quiescent hepatic stellate cells (HSCs) are marked by the expression of desmin and lecithin retinol acyltransferase (lrat) and function mainly as a reservoir for lipids and vitamin A. Once activated, HSCs trans-differentiate into myofibroblasts characteristically expressing smooth muscle actin (α-SMA) and periostin (Koyama and Brenner, 2017). Indeed, transcriptomic analyses reveal profound changes in gene expression patterns when quiescent HSCs become fully activated myofibroblasts in the fibrotic livers (Dobie et al., 2019; Marcher et al., 2019; Liu et al., 2020).

CXXC5 belongs to the CXXC domain zinc-finger family of proteins, comprising DNA methyltransferases, DNA demethylases, histone methyltransferases, and histone demethylases, that contribute to transcriptional regulation by preferentially binding to unmethylated CpG islands (Long et al., 2013). Recently, two independent investigations have implicated CXXC5 in the development of hepatocellular carcinoma (HCC), a pathology intimately associated with liver fibrosis. Yan et al. (2018) have reported that CXXC5 antagonizes HCC development by promoting TGF-β induced cell cycle arrest of liver cancer cells. On the contrary, Tan et al. (2018) have shown the CXXC5 promotes HCC malignancies by stimulating the proliferation, migration, and invasion of liver cancer cells. It remains to be determined whether and, if so, how CXXC5 may regulate the trans-differentiation of HSCs during liver fibrosis. Of note, Lee S.H. et al. (2015) have demonstrated that CXXC5-null mice display accelerated subcutaneous wound healing. Mechanistically, CXXC5 binds to the Dishevelled protein to promote β-catenin degradation thus blockading Wnt-mediated myofibroblast activation. We therefore hypothesized that CXXC5 might regulate HSC trans-differentiation. We report here that CXXC5 expression is down-regulated during HSC activation. On the contrary, CXXC5 over-expression antagonizes HSC activation. CXXC5 binds to the promoter region of MYCL1, a proto-oncogene, and represses MYCL1 transcription. Loss of CXXC5 leads to MYCL1 depression and HSC activation.

## Materials and Methods

### Animals

All the animal experiments were reviewed and approved by the Nanjing Medical University Ethics Committee on Humane Treatment of Laboratory Animals. To induce liver fibrosis, male C57BL/6 mice were injected with CCl_4_ (1.0 mL/kg as 50% vol/vol) or subjected to bile duct ligation (BDL) as previously described (Wang Q. et al., 2020; Li et al., 2021). Picrosirius red staining was performed with paraffin-embedded liver sections using a commercially available kit (Sigma Aldrich) as previously described (Li et al., 2021). For hydroxylproline quantification, ∼10 mg of liver tissue was weighed, homogenized, and hydrolyzed in HCl (10N) at 120°C for 3 h. Supernatants were transferred to a 96-well plate and hydroxylproline content was determined by colorimetry (Sigma Aldrich, MAK008).

### Cell Culture, Plasmids, and Transient Transfection

Immortalized human hepatic stellate cells (LX-2) were maintained in DMEM supplemented with 10% FBS. Primary hepatic stellate cells were isolated and maintained as previously described (Jin et al., 2020; Lv et al., 2020; Wu et al., 2020). Briefly, the animals were anesthetized by intraperitoneal injection with ketamine-xylazine. A laparotomy was performed and the portal vein was cut to allow retrograde perfusion with pronase (Sigma Aldrich, St. Louis, MO, United States) and collagenase (Roche, Germany) containing solutions. HSCs were isolated from the non-parenchymal fraction by 9.7% Nycodenz gradient centrifugation. Isolated HSCs were seeded in plastic culture dishes and allowed to undergo spontaneous activation. RNA targeting SRF (GAUGGAGUUCAUCGACAACAA) was purchased from Dharmacon. Recombinant TGF-β1 (100-21) was purchased from Peprotech. Transient transfections were performed with Lipofectamine 2000. Luciferase activities were assayed 24–48 h after transfection using a luciferase reporter assay system (Promega) as previously described (Yang et al., 2019a, b).

### Protein Extraction and Western Blot

Whole cell lysates were obtained by re-suspending cell pellets in RIPA buffer (50 mMTris pH7.4, 150 mMNaCl, 1% Triton X-100) with freshly added protease inhibitor (Roche) as previously described (Fan et al., 2020; Mao et al., 2020). Western blot analyses were performed with anti-CXXC5 (Cell Signaling Tech, 84546), anti-MYCL1 (Abcam, ab28739), anti-α-SMA (Abcam, ab5694), and anti-β-actin (Sigma, A1978). For densitometrical quantification, densities of target proteins were normalized to those of β-actin. Data are expressed as relative protein levels compared to the control group which is arbitrarily set as 1.

### RNA Isolation and Real-Time PCR

RNA was extracted with the RNeasy RNA isolation kit (Qiagen). Reverse transcriptase reactions were performed using a SuperScript First-strand Synthesis System (Invitrogen) as previously described. Real-time PCR reactions were performed on an ABI Prism 7500 system with the following primers: human *CXXC5*, 5′-CGGTGGACAAAAGCAACCCTAC-3′ and 5′-CGCTTCAGCATCTCTGTGGACT-3′; mouse *Cxxc5*, 5′-GTCC GAGCAGAGCCAGAAG-3′ and 5′-CGGCTGCCCACAATAGA GAT-3′; human *ACTA2*, 5′-CTATGCCTCTGGACGCACAACT-3′ and 5′-CAGATCCAGACGCATGATGGCA-3′; mouse *Acta2*, 5′-ACTGGGACGACATGGAAAAG-3′ and 5′-GTTCAGTGGT GCCTCTGTCA-3′; human *MYCL1*, 5′-CATGCAGTCACGG CGTATGAT-3′ and 5′-CTGCGGGGAGGATTTCTACC-3′; mouse *Mycl1*, 5′-TTCTACGACTATGACTGCGGA-3′ and 5′-TGATGGAAGCATAATTCCTGC-3′. Ct values of target genes were normalized to the Ct values of housekeekping control gene (18s, 5′-CGCGGTTCTATTTTGTTGGT-3′ and 5′-TCGTCTTCGAAACTCCGACT-3′ for both human and mouse genes) using the ΔΔCt method and expressed as relative mRNA expression levels compared to the control group which is arbitrarily set as 1.

### RNA Sequencing and Data Analysis

Total RNA was extracted using the TRIzol reagent according to the manufacturer’s protocol. RNA purity and quantification were evaluated using the NanoDrop 2000 spectrophotometer (Thermo Fisher Scientific, United States). RNA integrity was assessed using the Agilent 2100 Bioanalyzer (Agilent Technologies, Santa Clara, CA, United States). Then the libraries were constructed using TruSeq Stranded mRNA LT Sample Prep Kit (Illumina, San Diego, CA, United States) according to the manufacturer’s instructions and sequenced on an Illumina HiSeq X Ten platform and 150 bp paired-end reads were generated. Raw data (raw reads) of fastq format were firstly processed using Trimmomatic and the low quality reads were removed to obtain the clean reads. The clean reads were mapped to the mouse genome (Mus_musculus.GRCm38.99) using HISAT2. FPKM of each gene was calculated using Cufflinks, and the read counts of each gene were obtained by HTSeqcount. Differential expression analysis was performed using the DESeq (2012) R package. *P* value <0.05 and fold change >2 or fold change <0.5 was set as the threshold for significantly differential expression. Hierarchical cluster analysis of differentially expressed genes (DEGs) was performed to demonstrate the expression pattern of genes in different groups and samples. GO enrichment and KEGG pathway enrichment analysis of DEGs were performed respectively using R based on the hypergeometric distribution. The data presented in the study are deposited in the pubmed repository, accession number PRJRNA733841.

### Chromatin Immunoprecipitation

Chromatin Immunoprecipitation (ChIP) assays were performed essentially as described before (Coarfa et al., 2020; Maity et al., 2020; Wang J.N. et al., 2020; Wang S. et al., 2020; Marti et al., 2021). In brief, chromatin in control and treated cells were cross-linked with 1% formaldehyde. Cells were incubated in lysis buffer (150 mM NaCl, 25 mM Tris pH 7.5, 1% Triton X-100, 0.1% SDS, 0.5% deoxycholate) supplemented with protease inhibitor tablet and phenylmethylsulfonyl fluoride (PMSF). DNA was fragmented into ∼200 bp pieces using a Branson 250 sonicator. Aliquots of lysates containing 200 μg of protein were used for each immunoprecipitation reaction with anti-CXXC5 (Proteintech, 16513-1), anti-5′-methylcytosine (Abcam, ab214727), anti-acetyl H3K9 (Millipore, 07-352), anti-acetyl H3K27 (Millipore, 07-360), anti-RNA polymerase II CTD p-Ser2 (Abcam, ab138246), anti-RNA polymerase II CTD p-Ser5 (Abcam, ab252852), or pre-immune IgG. Precipitated DNA was amplified with the following primers: MYCL1 primer#1: 5′-ACCTGTCGACTGCCCGTAGTA-3′ and 5′-AGCCAGCACACACGCACAT-3′; MYCL1 primer#2, 5′-ATC TGTGTAGAAGATGAC-3′ and 5′-AGGGTCTCACTCTAGCG TCAA-3′; MYCL1 primer #3, 5′-ACATGGACTACGACTCGT AC-3′ and 5′-ACCCAAGCCCCAGGGCGGCGAC-3′; ITGB4 primer, 5′-CTCGGACAGTCCCTGCTC-3′ and 5′-GCTGCCG CTAGGAGATGG-3′.

### EdU Incorporation Assay

5-Ethynyl-2′-deoxyuridine (EdU) incorporation assay was performed in triplicate wells with a commercially available kit (Thermo Fisher Scientific) per vendor instruction. Briefly, the EdU solution was diluted with the culture media and added to the cells for an incubation period of 2 h at 37°C. After several washes with 1×PBS, the cells were then fixed with 4% formaldehyde and stained with Alexa Fluor^TM^ 488. The nucleus was counter-stained with DAPI. The images were visualized by fluorescence microscopy and analyzed with Image-Pro Plus (Media Cybernetics). For each group, at least six different fields were randomly chosen and the positively stained cells were counted and divided by the number of total cells. The data are expressed as relative EdU staining compared to the control group arbitrarily set as 100%.

### Statistical Analysis

One-way ANOVA with *post hoc* Scheffé analyses were performed by SPSS software (IBM SPSS v18.0, Chicago, IL, United States). Unless otherwise specified, values of *p*<0.05 were considered statistically significant.

## Results

### Down-Regulation of CXXC5 Expression Parallels Hepatic Stellate Cell Activation

**FIGURE 1:**
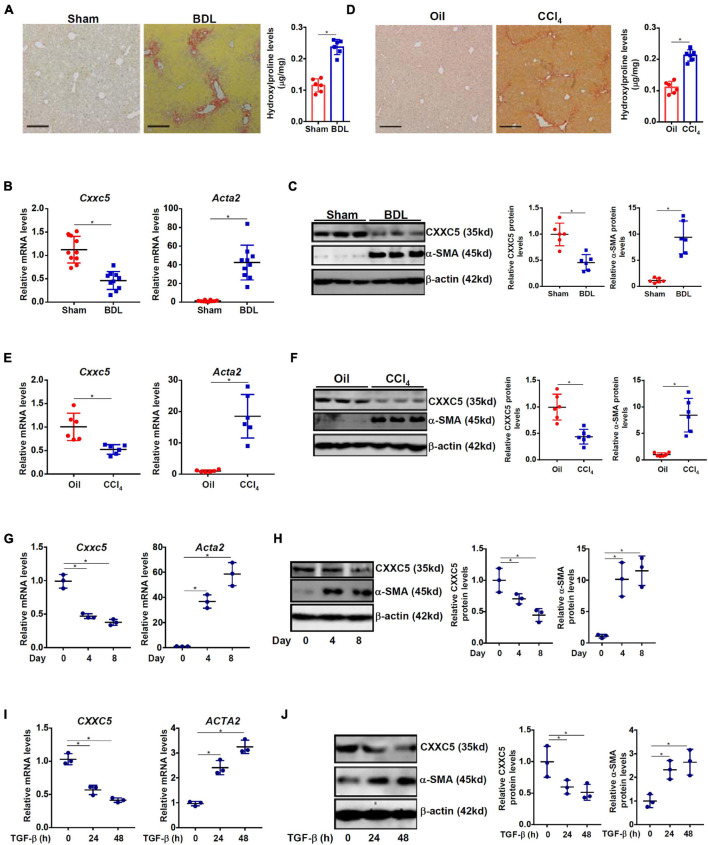
Down-regulation of CXXC5 expression parallels hepatic stellate cell activation. **(A–C)** C57/B6 mice were subjected to the BDL or the sham procedure for 2 weeks as described in section “Materials and Methods.” **(A)** Liver fibrosis was confirmed by picrosirius red staining and hydroxylproline quantification. Primary hepatic stellate cells were isolated and CXXC5 expression levels were examined by qPCR **(B)** and Western **(C)**. **(D–F)** C57/B6 mice were injected with or without CCl_4_ for 4 weeks as described in section “Materials and Methods.” **(D)** Liver fibrosis was confirmed by picrosirius red staining and hydroxylproline quantification. Primary hepatic stellate cells were isolated and CXXC5 expression levels were examined by qPCR **(E)** and Western **(F)**. *N* = 6–10 mice for each group. **(G,H)** Primary hepatic stellate cells were isolated from C57/B6 mice and allowed to undergo spontaneous activation in culture. CXXC5 expression levels were examined by qPCR and Western. **(I,J)** LX-2 cells were treated with or without TGF-β1 (2 ng/ml). The cells were harvested at indicated time points and CXXC5 expression levels were examined by qPCR and Western. All experiments were repeated three times and one representative experiment is shown. *p < 0.05.

We first evaluated the relationship between CXXC5 expression and HSC activation in different animal and cell models of liver fibrosis. In the first model liver fibrosis was induced in mice by BDL; liver fibrosis was evident as examined by picrosirius red staining (Figure 1A, left panel) and hydroxylproline quantification (Figure 1A, right panel). As shown in Figure 1B, mRNA expression of α-SMA (*Acta2*), a myofibroblast marker, was significantly up-regulated in primary HSCs isolated from the BDL mice compared to the sham mice indicative of HSC activation/trans-differentiation; accompanying *Acta2* up-regulation, there was a simultaneous down-regulation of *Cxxc5* mRNA. Western blotting showed that CXXC5 protein levels were also decreased in the primary HSCs isolated from the fibrotic livers compared to the normal livers (Figure 1C). In the second model in which the mice were injected with carbon tetrachloride (CCl_4_), robust liver fibrosis was detected by picrosirius red staining (Figure 1D, left panel) and hydroxylproline quantification (Figure 1D, right panel). Similarly, it was found that CXXC5 expression was down-regulated in the HSCs isolated from the fibrotic livers compared to the control livers (Figures 1E,F).

When primary HSCs were cultured *in vitro* and underwent spontaneous activation, there was a progressive elevation of *Acta2* expression and a simultaneous down-regulation of *Cxxc5* expression at both mRNA (Figure 1G) and protein (Figure 1H) levels. In human immortalized HSCs (LX-2), TGF-β1 treatment stimulated α-SMA expression but repressed CXXC5 expression (Figures 1I,J).

### CXXC5 Over-Expression Suppresses HSC Activation

**FIGURE 2:**
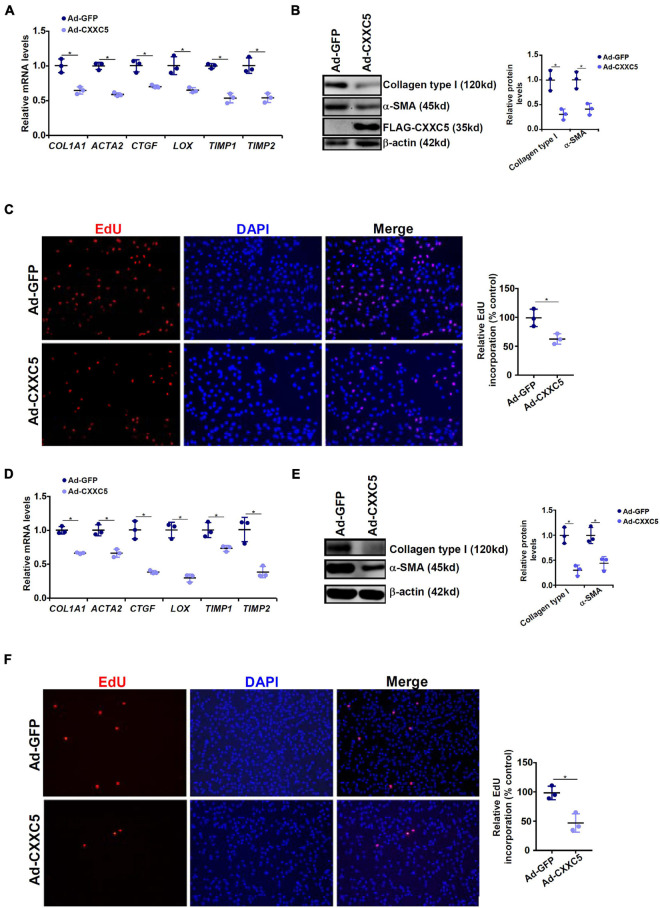
CXXC5 over-expression suppresses HSC activation. **(A–C)** LX-2 cells were infected with adenovirus carrying CXXC5 expression vector (Ad-CXXC5) or GFP (Ad-GFP). Gene expression levels were examined by qPCR and Western. Cell proliferation was measured by EdU staining. **(D–F)** Primary murine hepatic stellate cells were infected with adenovirus carrying CXXC5 expression vector (Ad-CXXC5) or GFP (Ad-GFP). Cell proliferation was measured by EdU staining. All experiments were repeated three times and one representative experiment is shown. *p < 0.05.

Because a negative correlation between CXXC5 expression and HSC activation was observed, we asked whether CXXC5 over-expression could impede HSC activation. LX-2 cells were infected with adenovirus carrying a FLAG-tagged CXXC5 expression vector or an empty vector. CXXC5 over-expression dampened the levels of several well-documented pro-fibrogenic genes including collagen type I, collagen type III, connective tissue growth factor, and tissue inhibitor of metalloproteinase (Figures 2A,B). In addition, CXXC5 over-expression suppressed HSC proliferation as assessed by EdU incorporation (Figure 2C). Similarly, CXXC5 over-expression down-regulated pro-fibrogenic gene expression (Figures 2D,E) and retarded proliferation rate (Figure 2F) in primary murine HSCs.

### CXXC5 Over-Expression Alters HSC Transcriptome

**FIGURE 3:**
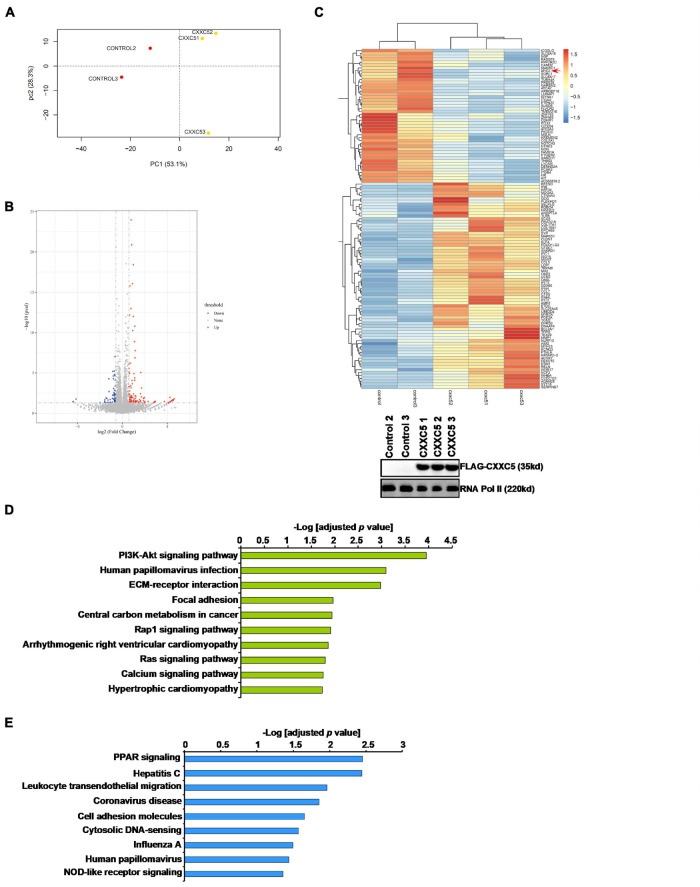
CXXC5 over-expression alters HSC transcriptome. LX-2 cells were infected with adenovirus carrying CXXC5 expression vector (Ad-CXXC5) or GFP (Ad-GFP). RNA-seq was performed as described in section “Materials and Methods.” **(A)** Sample clustering. **(B)** Volcano plot. **(C)** Heat map. Inset, CXXC5 over-expression levels were examined by Western blotting. **(D)** GO analysis. **(E)** KEGG analysis.

In order to identify potential target(s) of CXXC5 that could regulate HSC activation, we performed RNA-seq analysis comparing the transcriptome of LX-2 cells with CXXC5 over-expression and that of the control LX-2 cells. Principal component analysis (PCA) showed that CXXC5 over-expression significantly altered the transcriptome of LX-2 cells (Figure 3A). Using *p* value <0.05 and fold change >2 or fold change <0.5 as cutoffs, we were able to identify 58 genes down-regulated and 106 genes up-regulated by CXXC5 over-expression (Figures 3B,C). GO (Figure 3D) and KEGG (Figure 3E) analyses showed that several pathways related to liver fibrosis including PI3K-AKT signaling, ECM–receptor interaction, and focal adhesion were altered due to CXXC5 over-expression. Because many studies agree that CXXC5 primarily functions as a transcriptional repressor (Andersson et al., 2009; L’Hote et al., 2012), we focused on the genes repressed by CXXC5 over-expression. Several genes, including NCF (Rasi et al., 2007) and NOTCH3 (Chen et al., 2012), have been investigated for their roles in the pathogenesis of liver fibrosis. A definitive role for the others, including the proto-oncogene MYCL1, in HSC activation remains undetermined. Of note, qPCR verification confirmed that several other genes, including sortilin related receptor 1 (SORL1), protein tyrosine phosphatase non-receptor type 22 (PTPN22), integrin β4 (ITGB4), and sterile alpha motif domain containing 11 (SAMD11), were indeed repressed by CXXC5 over-expression in LX-2 cells (Supplementary Figure 1) but the relevance of these genes in HSC activation was not further investigated.

### MYCL1 Is a Novel CXXC5 Target

**FIGURE 4:**
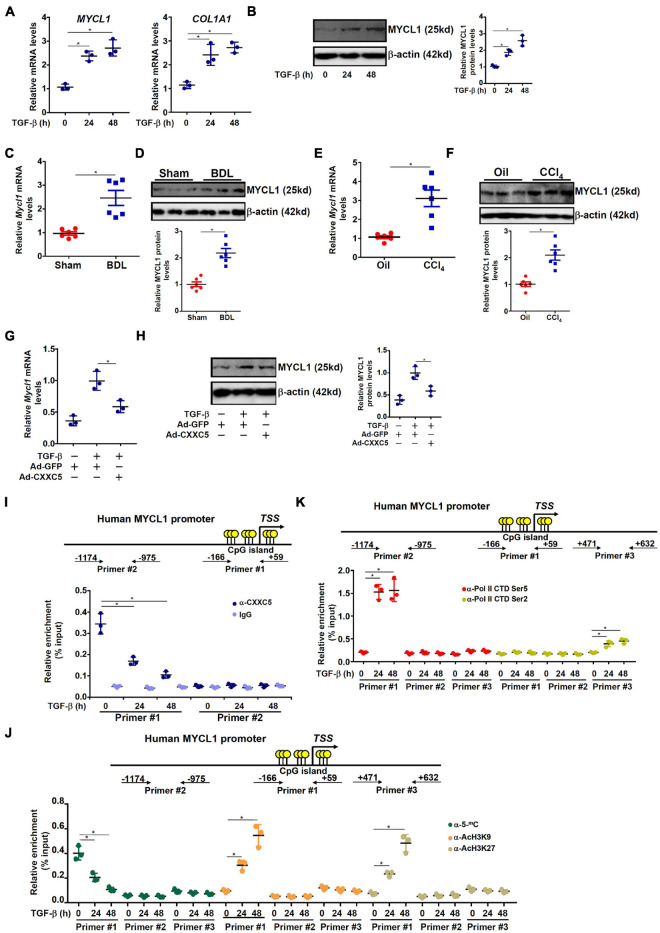
MYCL1 is a novel CXXC5 target. **(A,B)** LX-2 cells were treated with or without TGF-β1 (2 ng/ml). The cells were harvested at indicated time points and MYCL1 expression levels were examined by qPCR and Western. **(C,D)** C57/B6 mice were injected with or without CCl_4_ for 4 weeks as described in section “Materials and Methods.” Primary hepatic stellate cells were isolated and CXXC5 expression levels were examined by qPCR and Western. *N* = 6 mice for each group. **(E,F)** C57/B6 mice were subjected to the BDL or the sham procedure for 2 weeks as described in section “Materials and Methods.” Primary hepatic stellate cells were isolated and CXXC5 expression levels were examined by qPCR and Western. *N* = 6 mice for each group. **(G,H)** LX-2 cells were infected with infected with adenovirus carrying CXXC5 expression vector (Ad-CXXC5) or GFP (Ad-GFP) followed by TGF-β1 (2 ng/ml) treatment for 48 h. MYCL1 expression was examined by qPCR and Western. **(I)** LX-2 cells were treated with or without TGF-β1 (2 ng/ml). The cells were harvested at indicated time points and ChIP assay was performed with anti-CXXC5 or IgG. **(J)** LX-2 cells were treated with or without TGF-β1 (2 ng/ml). The cells were harvested at indicated time points and ChIP assay was performed with anti-5′-methylcytosine, anti-acetyl H3K9, or anti-acetyl H3K27. **(K)** LX-2 cells were treated with or without TGF-β1 (2 ng/ml). The cells were harvested at indicated time points and ChIP assay was performed with anti-RNA Pol II (ser5) and anti-RNA Pol II (ser2). All experiments were repeated three times and one representative experiment is shown. *p < 0.05.

C-MYC, the founding member of the MYC family of proto-oncogenes, has a well-established role in HSC activation and liver fibrosis (Potter et al., 1999; Nevzorova et al., 2013; Arteaga et al., 2016; Cai et al., 2018). In contrast, relatively little is known regarding the role of I-MYC (encoded by MYCL1 or MYCL), a closely related sibling of C-MYC. Therefore, we examined the regulation of MYCL1 expression by CXXC5 and its relevance in HSC activation. As shown in Figures 4A,B, MYCL1 expression was markedly induced by TGF-β1 treatment mirroring the down-regulation of CXXC5. MYCL1 expression, at both mRNA (Figure 4C) and protein (Figure 4D) levels, was higher in the activated HSCs isolated from the BDL mice than in the quiescent HSCs isolated from the sham mice. Similarly, it was also observed that MYCL1 expression was up-regulated in the HSCs isolated from the mice subjected to CCl_4_ injection compared to those isolated from the mice injected with corn oil (Figures 4E,F). On the contrary, over-expression of CXXC5 repressed the induction of MYCL1 expression by TGF-β1 treatment in LX-2 cells (Figures 4G,H). More importantly, ChIP assay showed that CXXC5 could directly bind to the CpG island region located surrounding the transcription start site (TSS) of the MYCL1 promoter; TGF-β1 treatment, however, severely dampened the occupancy of CXXC5 on the MYCL1 promoter (Figure 4I). By comparison, no binding of CXXC5 was detected on the ITGB4 promoter with no CpG island, suggesting that CXXC5 might regulate ITGB4 expression indirectly (Supplementary Figure 2). Of interest, removal of CXXC5 binding from the MYCL1 promoter upon TGF-β1 stimulation coincided with the erasure of DNA methylation (5′-methylcytosine) and accumulation of acetylated histone H3K9/H3K27 (Figure 4J) indicative of active chromatin remodeling. TGF-β1 treatment significantly augmented the enrichment of Pol II CTD Ser5 on the CXXC5 promoter region indicative of accelerated transcriptional initiation. There was a comparatively smaller but significant increase in the association of Pol II CTD Ser2 with the CXXC5 gene body, which may reflect secondarily enhanced elongation (Figure 4K).

### MYCL1 Promotes HSC Activation

**FIGURE 5:**
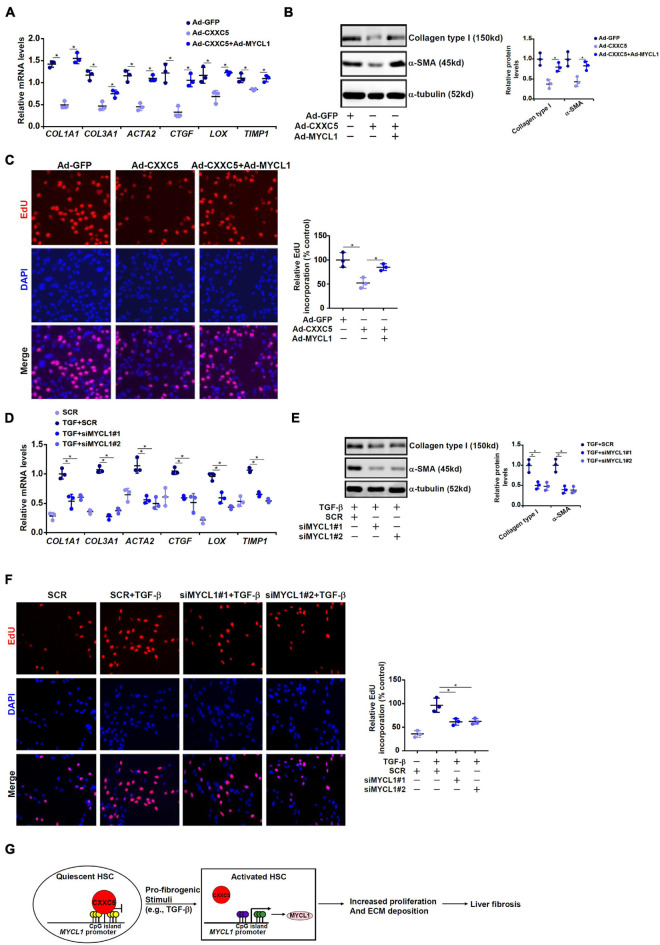
MYCL1 promotes HSC activation. **(A–C)** LX-2 cells were infected with indicated adenovirus. Gene expression levels were examined by qPCR and Western. Cell proliferation was measured by EdU staining. **(D–F)** LX-2 were transfected siRNA targeting MYCL1 or scrambled siRNA (SCR) followed by treatment with TGF-β1 (2 ng/ml). Gene expression levels were examined by qPCR and Western. Cell proliferation was measured by EdU staining. All experiments were repeated three times and one representative experiment is shown. **(G)** A schematic model. *p < 0.05.

We finally evaluated the contribution of MYCL1 to HSC activation *in vitro*. CXXC5 over-expression in LX-2 cells repressed the expression of pro-fibrogenic genes; simultaneous over-expression of MYCL1 reversed the suppression by CXXC5 and restored the expression of pro-fibrogenic genes (Figures 5A,B). In addition, MYCL1 over-expression normalized proliferation of LX-2 cells (Figure 5C), suggesting that MYCL1 could be placed downstream of CXXC5. On the contrary, TGF-β1 induced expression of pro-fibrogenic genes (Figures 5D,E) and cell proliferation (Figure 5F) could be attenuated by MYCL1 knockdown.

## Discussion

Expansion and trans-differentiation of quiescent hepatic stellate cells into mature myofibroblasts represent a hallmark event in liver fibrosis. Here we describe a novel role for the transcriptional regulator CXXC5 in HSC activation (Figure 5G). We show here that CXXC5 down-regulation correlates with HSC activation *in vivo* and *in vitro*. It remains unclear what mechanism may account for the repression of CXXC5 expression. Grimwade and colleagues have argued that promoter methylation of CXXC5 contributes to its down-regulation in patients with acute myeloid leukemia (AML; Kuhnl et al., 2015). Because all three isoforms of DNA methyltransferases (DNMTs) are activated during HSC trans-differentiation (Gotze et al., 2015), it is reasonable to postulate that CXXC5 repression may result from DNMT-mediated hypermethylation. Alternatively, Yasar et al. (2016) have reported that estradiol, through estrogen receptor (ER), activates CXXC5 transcription in breast cancer cells. Since the E2-ER axis plays an inhibitory role in HSC activation and liver fibrosis (Zhang et al., 2018), it is tempting to speculate that CXXC5 repression may be secondary to dampened E2-ER signaling.

Although we provide data to show that CXXC5 over-expression antagonizes HSC trans-differentiation, the *in vivo* relevance of this finding remains to be ascertained. CXXC5-null mice are viable and display no other overt phenotypes than enlargements of the skull, scapula, spine, ribs, and limb bones (Kim et al., 2015), suggesting that CXXC5 is unlikely a major contributor to hepatic homeostasis under physiological conditions. On the other hand, Cheng et al. (2020) have demonstrated that CXXC5 knockdown, achieved by adenovirus carrying CXXC5-targeting shRNA, attenuates bleomycin-induced pulmonary fibrosis in mice possibly owing to increased apoptosis of lung fibroblasts although the specificity of this system remains questionable. More recently, it has been shown that CXXC5 regulates the phenotype of plasmacytoid dendritic cells (pDCs) by functioning as a transcriptional repressor of the pro-inflammatory protein IRF7 (Ma et al., 2017). Of interest, Wu et al. (2019) have reported that IRF7 bridges inflammation to fibrogenesis by interacting with SMAD3, a key mediator of TGF-β signaling and that IRF7 knockout mice are protected from systemic sclerosis, a prototypical form of inflammation-associated tissue fibrosis. Because CXXC5 deficiency leads to IRF7 up-regulation in mice (Ma et al., 2017), which may potentially trigger a pro-fibrogenic response, it would be of great help to examine the phenotype of the CXXC5-null mice in the settings of the liver fibrosis. Although our data indicate that CXXC5 may regulate proliferation and expression of pro-fibrogenic genes in HSCs, other potential mechanisms are not fully examined. For instance, it has been proposed that accelerated apoptosis of HSCs may be considered as an approach to mitigate or reverse liver fibrosis (Elsharkawy et al., 2005). Previous studies have shown that CXXC5 promote apoptosis in neurons and cancer cells by amplifying the TGF-β-SMAD pathway (Wang et al., 2013; Yan et al., 2018). Therefore, it is possible that CXXC5 may regulate HSC phenotype by skewing the death-survival balance.

Through RNA-seq, we have identified the proto-oncogene MYCL1 as a direct transcriptional target for CXXC5. Further, we demonstrate here that MYCL1 activates expression of pro-fibrogenic genes and promotes cell proliferation in HSCs. MYCL1 belongs to the myelocytomatosis (MYC) family of multifaceted transcription regulators sharing significant homology with c-MYC and MYCN (Bragelmann et al., 2017). Prior to our investigation, no evidence existed to link MYCL1 to HSC activation and/or liver fibrosis. Mounting evidence suggests that MYCL1 hyperactivation is associated with augmented proliferation in malignant cancer cells (Diolaiti et al., 2015; Suzuki et al., 2016; Kamibeppu et al., 2018). For instance, MacPherson and colleagues have discovered that lung cancer cells originated from MYCL1-null mice exhibited slower proliferation than those from the wild type mice and are unable to form malignant tumors (Kim et al., 2016). In addition, targeted inhibition of cancer cell proliferation appears to correlate with down-regulation of MYCL1 expression (Kato et al., 2016). However, no consensus has emerged from previous studies with regard to the exact mechanism whereby MYCL1 regulates cellular proliferation. Based on RNA-seq analysis of lung cancer cell transcriptome driven by MYCL1 over-expression, Kim et al. (2016) have proposed that MYCL1 may stimulate cell proliferation by orchestrating RNA polymerase I-dependent ribosomal RNA synthesis. There is vidence to suggest that the rRNA synthesis pathway is involved in HSC activation (Lechuga et al., 2004; Yang et al., 2009; Morales-Ibanez et al., 2016). Presumably MYCL1 contributes to HSC proliferation via a similar mechanism but this hypothesis clearly warrants further investigation. Recently, Wakao et al. (2017) have presented evidence that over-expression of MYCL1 in human fibroblasts induces a muscle-like phenotype. This observation provides support for our finding that MYCL1 may be involved in the acquisition of a contractile phenotype characteristic to HSC trans-differentiation although the mechanism is not clear. Similar to CXXC5, MYCL1 deficiency in mice is compatible with embryogenesis suggesting that MYCL1, like CXXC5, is not required to maintain the quiescence of the HSC population (Hatton et al., 1996). It would be of high interest to determine how MYCL1 deletion would influence liver fibrosis in mice.

Our data show that de-repression of MYCL1 transcription by CXXC5 down-regulation is associated with erasure of DNA methylation and accumulation of histone acetylation on the MYCL1 promoter. Tsuchiya et al. (2016) have reported that CXXC5 can interact with the H3K9 trimethyltransferase SUV39H1 in T lymphocytes to repress Cd40lg transcription. On the one hand, a coordination between SUV39H1-dependent H3K9 trimethylation and DNA methylation is well documented (Cedar and Bergman, 2009). Thus, it is conceivable that CXXC5 may recruit SUV39H1 to the MYCL1 promoter, which consequently enlists DNMTs to catalyze CpG methylation. In addition, because H3K9 methylation is antagonistic to H3K9 acetylation, removal of CXXC5 from the MYCL1 may dampen SUV39H1 recruitment and H3K9 methylation, which may secondarily up-regulate H3K9 acetylation. On the other hand, CXXC5 can directly interact with histone deacetylase 1 (HDAC1) to regulate the TGF-β signaling (Yan et al., 2018). It is possible that departure of CXXC5 from the MYCL1 promoter steers away the HDACs and histone acetylation then increases as a result. Clearly, further elucidation of the detailed epigenetic mechanism underlying MYCL1 trans-activation may shed additional light on the role of CXXC5 as a regulator of HSC trans-differentiation.

In summary, our data identify a CXXC5-MYCL1 axis that contributes to HSC activation. Screening for small-molecule compounds that boost CXXC5 activity could potentially yield novel therapeutic strategies in the intervention of liver fibrosis.

## Data Availability Statement

The original contributions presented in the study are publicly available. These data can be found in the PUBMED BioProject database, accession number PRJRNA733841.

## Ethics Statement

The animal study was reviewed and approved by Nanjing Medical University Ethics Committee on Humane Treatment of Experimental Animals.

## Author Contributions

XS and WZ conceived the project, secured funding, and provided supervision. XW, WD, and MK designed the experiments. XW, WD, MK, HR, JW, LS, and ZZ performed the experiments and collected the data. All authors wrote the manuscript.

## Conflict of Interest

The authors declare that the research was conducted in the absence of any commercial or financial relationships that could be construed as a potential conflict of interest.

## Publisher’s Note

All claims expressed in this article are solely those of the authors and do not necessarily represent those of their affiliated organizations, or those of the publisher, the editors and the reviewers. Any product that may be evaluated in this article, or claim that may be made by its manufacturer, is not guaranteed or endorsed by the publisher.
