# Supplementary material for: Down-Regulation of CXXC5 De-Represses MYCL1 to Promote Hepatic Stellate Cell Activation
Source: Front Cell Dev Biol. 2021 Sep 21;9:680344. doi: 10.3389/fcell.2021.680344 (PMC8490686; doi:10.3389/fcell.2021.680344)
Supplement: Supplementary file 1 [file Data_Sheet_1.docx]

**Supplementary material**

**Figure S1:** LX-2 cells were infected with adenovirus carrying CXXC5 expression vector (Ad-CXXC5) or GFP (Ad-GFP). Gene expression levels were examined by qPCR and Western.

**Figure S2:** LX-2 cells were treated with or without TGF-β1 (2ng/ml). The cells were harvested at indicated time points and ChIP assay was performed with anti-CXXC5 or IgG.
